# Investigating task preparation and task performance as triggers of the backward inhibition effect

**DOI:** 10.1007/s00426-022-01780-x

**Published:** 2022-12-26

**Authors:** Laura Joy Prosser, Motonori Yamaguchi, Rachel Swainson

**Affiliations:** 1 School of Psychology, University of Aberdeen, Aberdeen, UK; 2 Department of Psychology, University of Essex, Colchester, UK

## Abstract

Backward inhibition is posited to aid task switching by counteracting the tendency to repeat a recent task. Evidence that factors such as cue transparency affect backward inhibition seems to imply that it is generated during task preparation, making its absence following trials on which a prepared task was not performed (nogo trials) surprising. However, the nogo method used in previous studies might have prevented detection of preparation-driven effects. We used a truncated-trial method instead, omitting stages of a trial with no need for a nogo signal. In Experiment 1, an *n* − 2 repetition cost (suggested to indicate backward inhibition) followed trials truncated after response selection, indicating that response execution is not necessary to trigger backward inhibition. In Experiments 2 and 3, no *n* − 2 repetition cost was obtained following trials truncated after cue presentation. To ensure some task preparation on cue-only trials, Experiment 4 used a double-registration procedure where participants responded to the task cue and the target on each trial. In contrast to Experiments 2 and 3, a small *n* − 2 repetition cost followed trials truncated after cue responses, affecting cue responses on the current trial. In addition, the *n* − 2 repetition cost was increased at cue responses and became evident at target responses when the preceding trial also involved a target response. These results imply that backward inhibition might be generated by processes occurring up to and including a cue response, affecting subsequent cue responses, as well as during task performance itself, affecting subsequent cue and target responses.

**Supplementary Information:**

The online version contains supplementary material available at 10.1007/s00426-022-01780-x.

## Introduction

The environment that we live in is complex, providing a multitude of options for actions that can be performed. Our goals also shift frequently, such that what is an appropriate action from one moment to the next differs. Mayr and Keele (2000) proposed that there is a cognitive control mechanism that enables us to shift more effectively from one goal to the next. This mechanism, known as *backward inhibition*, is thought to decrease the amount of competition arising from the previous goal/task by inhibiting it when we try to perform a new task, to prevent the previous task from being performed again by mistake. This article asks when backward inhibition is triggered: is it triggered proactively, in advance of difficulty resulting from shifting goals, or reactively, in response to difficulty resulting from shifting goals?

Braver and colleagues proposed two distinct modes of cognitive control (Braver, 2012; Braver et al., 2007) that make up the Dual Mechanisms of Control framework. One mode is reactive control, which involves control being assigned when it is needed, at the point at which interference is detected. The other mode of control is proactive control, which is where attention is assigned prior to the need for it. For cognitive control to be reactive there needs to be conflict that is detected, whereas for proactive control there needs to be a contextual task cue that causes goal activation and goal maintenance. Within a backward inhibition task switching paradigm (as was used in this set of experiments), cognitive control would apply a reactive mechanism to dampen conflict that arises from the sharing of responses between tasks (i.e. using the left and right response button for all three tasks) or the use of trivalent stimuli (i.e. stimuli which allow a response from all three tasks), for example. Within the same paradigm, proactive control would be driven by processing of the task cue which occurs on the screen prior to a target being presented. The task cue indicates which task participants must perform on the upcoming target and therefore processing of this task cue should cause goal activation (i.e. the selection of a task) and goal maintenance (continued selection of the task), which Braver and colleagues suggested results in proactive control.

Mayr and Keele (2000) measured backward inhibition by comparing performance on the final trial of two three-trial sequences, ABA and CBA (where A, B and C refer to different tasks). They found that responses were slower on trial *n* (the final trial) of the ABA trial sequences relative to the CBA trial sequences; this difference is known as the *n* − 2 repetition cost. Mayr and Keele suggested that this cost is caused by the inhibition put in place on trial *n* − 1, to prevent interference from the task completed on trial *n* − 2, needing to be overcome on ABA sequences but not CBA sequences. In Experiment 3, they removed irrelevant distractors from the target screen, so that only one task could be performed on each trial. Although participants could perform the task correctly without a task cue, the researchers still presented task cues and instructed participants to use them explicitly in one condition, whereas participants were not presented with task cues in the other condition. They found an *n* − 2 repetition cost in the former condition but not in the latter, indicating that backward inhibition depends on proactive control. We also found similar results (Prosser et al., 2020), supporting the idea that backward inhibition is triggered proactively.

Several other studies have provided indirect evidence for backward inhibition being triggered proactively. In studies by Astle et al. (2012) and by Costa and Friedrich (2012), finding backward inhibition with stimuli that afforded a response in only one task led the authors to propose that task cues were the most likely trigger. Several studies reported that less transparent (more arbitrary) cue-task relationships were associated with higher *n* − 2 repetition costs (Arbuthnott, 2005; Arbuthnott & Woodward, 2002; Gade & Koch, 2014; Houghton et al., 2009); the dependency of the size of backward inhibition on an aspect of the task cues in these studies implies that the cue itself might be the trigger for the application of inhibition. [Also see Kuhns et al., (2007), and Hübner et al., (2003), for evidence of inhibition of the previous task being triggered prior to target processing in a flanker-compatibility-effect design.]

If backward inhibition is a form of proactive control, it would be triggered prior to target onset, so it should be evident even when target processing was not required on the preceding trial. However, the studies by Schuch and Koch (2003) and by Philipp et al. (2007) appear to provide evidence against this prediction. In these studies, participants used a go/nogo tone to decide whether they should respond to the target on each trial. Schuch and Koch’s study presented a go or nogo tone simultaneously with the target, so a nogo tone signalled that participants did not need to process the target or select a response. Hence, on nogo trials, both response selection and response execution did not occur. Philipp et al.’s study presented a go or nogo tone 100 or 1500 ms after the target onset. They suggested that 1500 ms would have been sufficient for response selection to have taken place before the tone. Hence, on nogo trials, response selection, but not response execution, had taken place. Importantly for our purposes, in both of those studies the nogo tone happened after task preparation would have occurred, so if backward inhibition had been triggered proactively during cue processing, an *n* − 2 repetition cost should presumably still have been seen following the nogo trials. In both cases, however, there was no significant *n* − 2 repetition cost following nogo trials. Schuch and Koch concluded that backward inhibition was not triggered if response selection did not occur; Philipp et al. concluded that response execution rather than response selection was critical to triggering the effect. Both of these papers argue in favour of backward inhibition being a form of reactive control, for instance being applied to overcome conflict related to response processing (see also Koch et al., 2010). On the face of it, the results of these studies appear to argue against backward inhibition being triggered proactively.

However, there might be a problem with drawing such a conclusion from these studies that used nogo trials because it might be that the presentation of a nogo tone eliminated backward inhibition triggered by task preparation, rather than the exclusion of response selection or execution stages of task processing preventing it from being triggered. Lenartowicz et al. (2011) used what we call a *truncated-trial* method and compared it to the go/nogo method in a study of task-switching (between two tasks). They replaced nogo trials with *cue-only* trials, in which participants were presented only with a task cue, but without a target stimulus. Thus, cue-only trials truncated a trial rather than informing the participant to stop as on nogo trials. Lenartowicz et al. examined the switch cost (i.e. the increase in RT on BA versus AA trial sequences), rather than the *n* − 2 repetition cost, and they found that there was no significant switch cost when the previous trial was a nogo trial, but there was still a significant switch cost when the previous trial was a cue-only trial. The researchers suggested that the nogo signal might have interfered with the effects of task preparation and abolished the subsequent switch cost.

We reasoned that if a nogo signal can abolish the switch cost by interfering with the effects of preparation that occurred before the nogo signal, then it might have abolished the *n* − 2 repetition cost in Schuch and Koch’s (2003) and Philipp et al.’s (2007) studies as well, by interfering with the effects of processes occurring before the nogo signal was presented. If so, it should be possible to observe backward inhibition following a truncated trial on which the same stages of task processing were removed as were excluded on the nogo trials of those previous studies. Such results would support the claim that backward inhibition is a form of proactive control if the critical stage was (or included) task preparation. Therefore, the present studies used a truncated-trial methodology to eliminate various stages of task processing in four experiments and examined whether backward inhibition is triggered by processing that occurs during task preparation or during task performance.

**Fig. 1:**
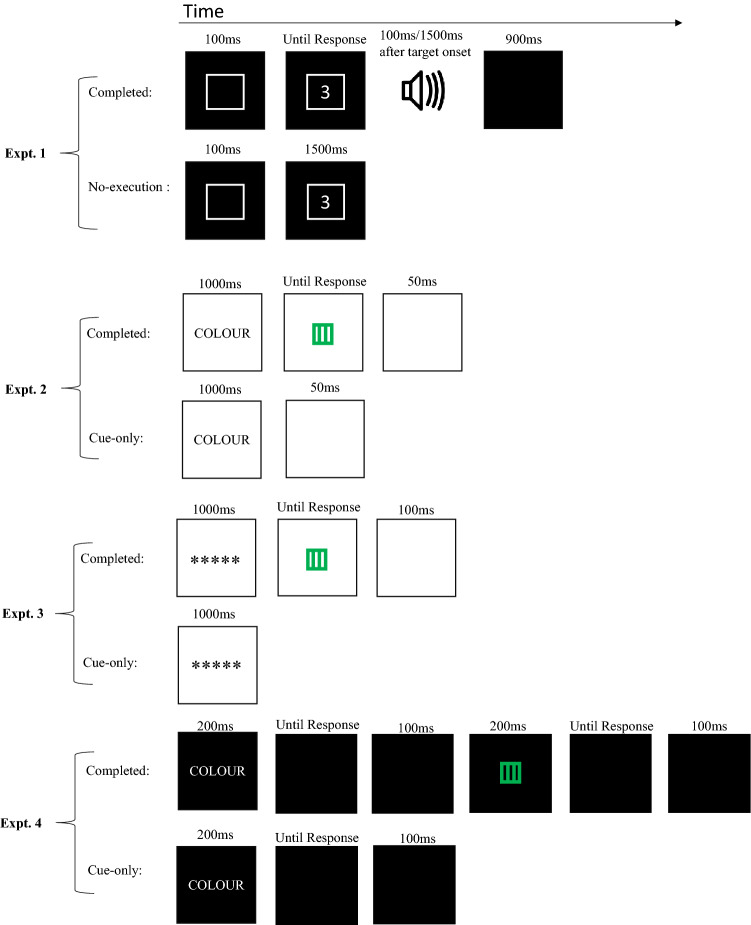
Trial sequences for Experiments 1, 2, 3, and 4. The background of Experiment 1 was black, Experiment 2 and 3 was white and Experiment 4 was dark grey (20, 20, 20 RGB). On Experiment 1 completed trials, the target was presented on the screen and then either 100 or 1500 ms after the target was presented the response signal (auditory tone) was played. The target stayed on the screen until a response was given

Experiment 1 tested whether response execution was necessary for backward inhibition by truncating trials after response selection. Philipp et al. (2007) presented a go or nogo tone informing participants to respond or not to respond to the target. In our experiment, on trials requiring a response we presented a go tone informing participants to respond to the target (*completed trials*), and on trials requiring no response we simply ended the trial without a go tone (*no-execution trials*) and therefore with no need to present a nogo tone (see Fig. 1). Experiments 2 and 3 investigated whether the preparation stage of task processing could trigger backward inhibition by truncating trials after a task cue. In contrast to Experiment 1, these experiments omitted a target (*cue-only trials*) as in Lenartowicz et al.’s (2011) study. In Experiment 4, we used a double-registration design (Arrington et al., 2007), in which participants responded both to task cues (cue response) and to targets (target response). On cue-only trials, this ensured that task cues were processed to the level of task identification. This experiment also allowed us to observe the effects of backward inhibition upon task preparation and task performance separately.

At this point, it is important to note that while the *n* − 2 repetition cost was initially put forward as providing evidence for an inhibitory mechanism, whether inhibition necessarily underlies the effect has been questioned in recent years (Gade et al., 2017; Grange et al., 2017). It may be that the effect is partly, or even completely, due to an *episodic mismatch* effect. That is, it might be that returning to a recent task but with different trial features (as would usually be the case on a proportion of ABA trials) is particularly difficult because the episodic memory trace for the previous event interferes with processing of information for the current trial. In the experiments described here, we do not attempt to distinguish possible causes of the *n* − 2 repetition cost but instead simply aim to determine which trial events are able/required to trigger it. Thus, we cannot rule out the possibility that the *n* − 2 repetition cost that we use as our measure of backward inhibition may, at least in part, be measuring an interference effect rather than an inhibition effect. We return to this issue below.

## Analysis plan

The four experiments were designed to ask similar questions and therefore we have conducted the same analysis within each. Our main question in each experiment was whether the stages of task processing remaining on the truncated trials (no-execution trials in Experiment 1; cue-only trials in Experiments 2–4) were sufficient to trigger backward inhibition that could be observed on the subsequent trial. We addressed this question by running a one-tailed *t* test on data from trials following truncated trials to see if ABA trial sequences had worse performance than CBA sequences (i.e. an *n* − 2 repetition cost). To show that the experimental setup could reliably produce backward inhibition, we also tested for backward inhibition following completed trials, again with a one-tailed *t* test. This would be especially important for the interpretation of any absent *n* − 2 repetition cost following truncated trials.

It has been suggested that backward inhibition is a flexible mechanism that is applied to the aspects of the task context that cause interference (Arbuthnott, 2008, 2009; Houghton et al., 2009) and so it could be that if there are multiple areas of interference the strength of backward inhibition triggered might increase as additional stages of task processing are completed. Therefore, we ran a further one-tailed *t* test to examine if the *n* − 2 repetition cost following completed trials was larger than the *n* − 2 repetition cost following the truncated trials.

For Experiments 3 and 4, we preregistered some or all of these comparisons (see “Data availability” section). With hindsight, these preregistered analyses do not seem optimal: specifically, the preregistration for Experiment 3 did not specify that the test for the *n* − 2 repetition effect should be one-tailed (see the “Method” for details) and the preregistration for Experiment 4 did not include a comparison of the size of the *n* − 2 repetition costs between completed and cue-only trials. We have chosen to present the results as outlined above for all four experiments to directly and consistently address our key questions. The deviation from the preregistration is reported in the relevant “Results” section, and the additional tests of our key hypotheses that were not preregistered are clearly described as such in the relevant “Results” section.

Finally, to explore the data further, we also ran a conventional repeated-measures analysis of variance (ANOVA, not preregistered) in all experiments, with the factors of trial sequence (ABA, CBA) and previous trial completion (truncated, completed), and with two-tailed *t* tests for analysis of simple main effects of trial sequence where the interaction between trial sequence and previous trial completion was significant. We note that while the ANOVA method provides a potentially useful view of the overall pattern of results, it does not provide a direct test of our key questions because it would not allow us to test for the trial-sequence effect following just cue-only trials unless a significant interaction had first been found. In addition, while tests of the interaction effect in the ANOVA and the follow-up tests of simple main effects might appear to be completely equivalent to the one-tailed *t* tests, this is not the case because the former tests are not directional, so only half of their statistical power is available to test our hypotheses. In contrast, the one-tailed *t* tests look for specific, directional effects that can directly answer our research questions: whether there is a *cost* of *n* − 2 task repetition following either cue-only or completed trials; and whether there is an *increase* in cost following completed versus cue-only trials.

## Experiment 1

This experiment examined whether response execution is necessary to trigger backward inhibition, as suggested by Philipp et al., (2007, Experiment 2). Philipp et al. allowed response selection but not response execution to take place on nogo trials. We wished to do the same but without involving any nogo element. To achieve this, we truncated trials (see Lenartowicz et al., 2011) after the response selection stage by simply not presenting the post-target tone that would otherwise inform participants to execute the selected response on these trials. These *no-execution* trials ended 1500 ms after target onset without a response and replaced Philipp et al.’s nogo trials. In the remaining *completed* trials, we used a tone (*response signal)* as these trials required a response, which is equivalent to Philipp et al.’s go trials. A response signal occurred after a delay of either 100 or 1500 ms from target onset and participants were instructed not to respond prior to hearing the response signal. As a consequence, we had three types of trial: no-execution trials (no response signal) and two types of completed trials (with 100 and 1500 ms response-signal delays). To have a sensitive measure of backward inhibition, as Philipp et al. did to explore the effects of response execution in the previous trial, we only analysed completed trials with a 100 ms response-signal delay that followed either no-execution trials or 1500 ms response-signal-delay completed trials.

As no-execution trials removed response execution processes but retained all processes preceding response execution, we would expect to only see an *n* − 2 repetition cost following completed trials, but not following no-execution trials, if response execution generates backward inhibition (Philipp et al., 2007). However, if processes prior to response execution generate backward inhibition, an *n* − 2 repetition cost should be obtained following both completed trials and no-execution trials. If the latter is the case, it would imply that nogo signals were abolishing backward inhibition triggered prior to response execution in Philipp et al.'s (2007) study.

### Methods

#### Participants

Fifty-one participants were tested in total for either course credit or £7 compensation for their time. Participants’ overall accuracy rate had to be above 70% (Arbuthnott & Woodward, 2002) and each participant had to have fewer than 10% of experimental trials removed (Los, 1999) due to the response time being below 200 ms or above 2500 ms to be included in the analysis. Therefore, two participants were excluded for accuracy and nine participants were excluded for response times. Of the 40^1^ remaining participants, 31 were female and their age ranged from 18 to 35 years (mean age: 19.73 years).

#### Apparatus and stimuli

The stimuli and tasks used were based on Philipp et al.'s (2007) Experiment 2. The target stimuli consisted of the digits 1–9 excluding 5. Participants had to decide whether the digit was greater or less than 5 (magnitude task), whether it was odd or even (parity task) or whether the digit was located centrally (3, 4, 6, 7) or peripherally (1, 2, 8, 9) in the interval from 1 to 9 (number line task). Each target was white on a black background with a frame around the digit. The frame served as the task cue. It was also white and had a shape of a diamond for the magnitude task, triangle for the number line task or a square for the parity task. A beep tone (600 Hz played for 200 ms) indicated when a response was required. Participants made responses using their left index finger (left response button) and right index finger (right response button) on a Cedrus button box (Cedrus Corporation, 2003). The eight possible response mappings were counterbalanced across all tested participants.

The following restrictions were based on the design of Philipp et al.’s (2007) Experiment 2. The sequence of trials was controlled, with equal numbers of each task and category (i.e. same number of odd trials as even trials), each response-signal delay on completed trials and each task sequence (ABA and CBA). Task repetitions were prevented. Repetition of a target stimulus was avoided in consecutive trials. No-execution trials (trials truncated after response selection) did not occur consecutively. Additionally, the stimulus that was last presented with one task could not be presented the next time that task occurred.

#### Procedure

Participants were informed of the category-response key mappings orally and visually, with a sheet depicting the mappings placed in front of them for the whole experiment. Participants sat at a comfortable distance from the computer which was running E-Prime 2.0 software (Psychology Software Tools, Inc., http://www.pstnet.com). Participants started with a practice block of 19 trials, which involved switching between all three tasks and included no-execution trials. The experiment itself consisted of 14 blocks of 84 trials each. In Philipp et al.’s design, 25% of all trials were nogo trials, and the ratio of 100 and 1500 ms signal delay for both go and nogo trials was 50:50. We chose not to include no-execution trials with a 100 ms signal delay, because with the truncated-trial method these were extremely short trials and proved to be confusing. Hence, the ratio of numbers of trials in each of the three trial types, no-execution: 1500 ms-delayed-response-completed: 100 ms-delayed-response-completed, was 1:3:3.

As shown in Fig. 1, each trial started with the presentation of a task cue for 100 ms. A digit was then presented within the task cue frame. If the trial was a completed trial, then after a 100 or 1500 ms response-signal delay an auditory tone was played, informing participants to respond. (It should be noted the response time was recorded from the onset of the response-signal rather than the onset of the target). Once participants responded, the stimuli (task cue frame and digit) were removed from the screen. A blank screen was then presented for 900 ms before the start of the next trial. If the trial was a no-execution trial then 1500 ms after target presentation the next trial started immediately, with the presentation of the task cue. (No tone was played on no-execution trials.)

If a wrong response was given, or a response given when it should not have been (i.e. on a no-execution trial or before the response-signal), feedback was then presented for 500 ms in the centre of the black screen in magenta (“WRONG ANSWER”, “WAIT FOR THE BEEP”). Response time (RT) was recorded on completed trials as the interval between the onset of a response signal and the pressing of a response key.

### Results

#### Data processing and analysis plan

Mean RT and percentage of trials with an error (PE) were computed for each participant for completed trials with 100-ms response delay that followed a no-execution trial and that followed a completed trial with 1500-ms response delay. We only included trials where trial *n* − 2 was completed (i.e. either 100 or 1500 ms-delayed-response trials) to ensure that previous no-execution and previous completed trial sequences only differed with respect to trial *n* − 1.

Trial exclusions were as follows: the first two trials of every block were excluded from analysis; trial sequences were excluded if trials *n* − 2 and *n* were not both completed trials; if the response of either of the previous two trials (*n* − 2 and *n* − 1) was inaccurate then that trial (*n*) was excluded; and for the RT analysis the current completed trial was excluded if the response was incorrect (4.26% of all completed trials in Experiment 1 were excluded because the current response was incorrect). Additionally, trials were excluded if RT was below 200 ms or above 2500 ms (4.35% of all completed trials in Experiment 1), as was any trial where a response was given prior to the response signal being played (0.77% of all trials). For the RT analysis, after exclusions there were on average 36 trials per trial sequence (ABA, CBA) per participant for previous no-execution trial sequences and on average 72 trials for previous completed trial sequences.

**Fig. 2:**
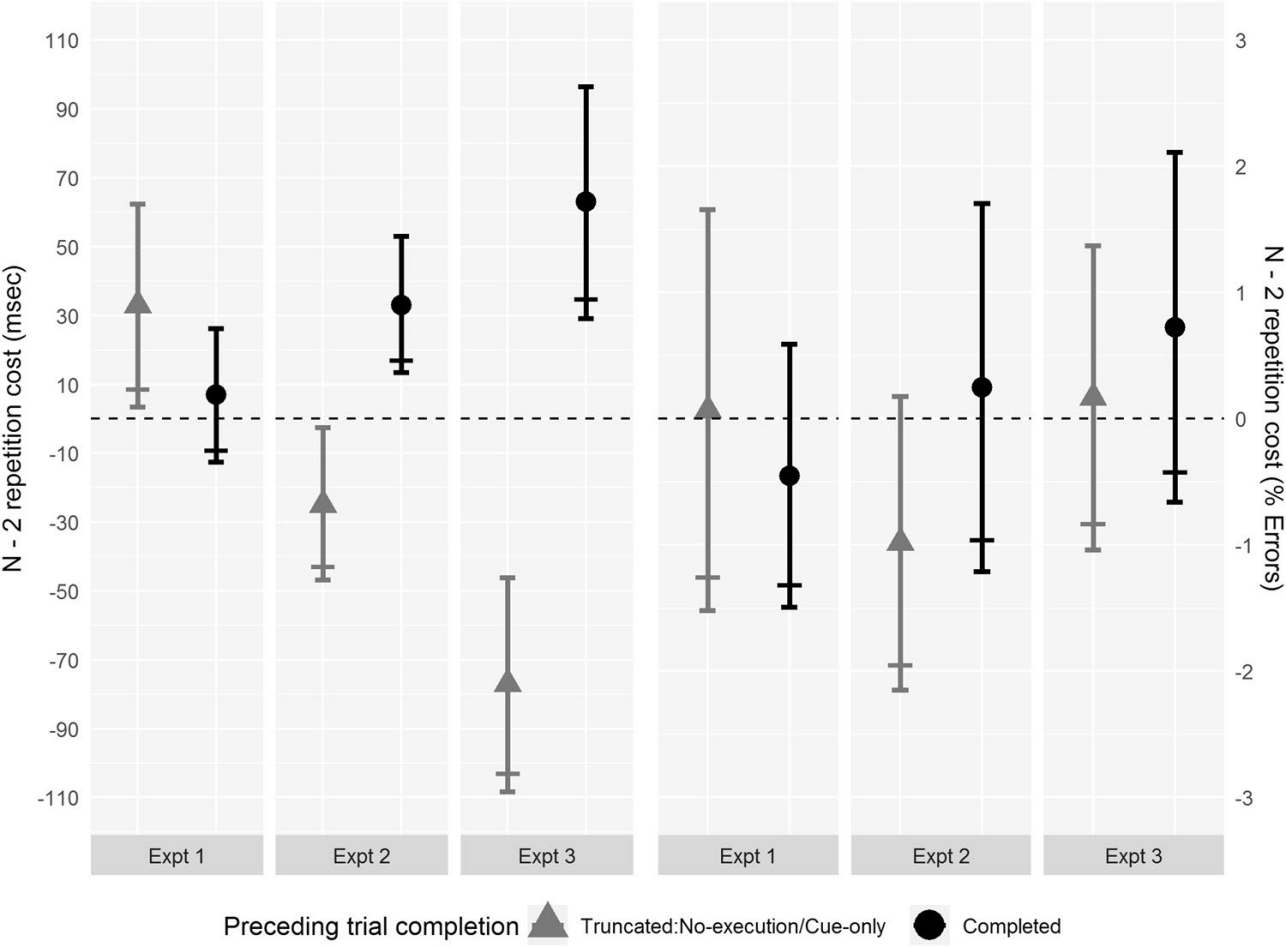
Experiments 1, 2 and 3: *n* − 2 repetition cost. Left side of the graph is RT data. Right side of the graph is % error data. Grey triangles show data from completed trials following truncated trials: no-execution for Experiment 1, cue-only for Experiments 2 and 3. Black circles show data from completed trials following completed trials. Narrow error bars above and below means show 95% CIs. Wide error bars below means show lower limit of 90% CI which indicate whether our key analysis of the *n* − 2 repetition costs are significant; where this bar is above the dashed zero line, the one-tailed *t* test for the presence of an *n* − 2 repetition cost is significant (*p* < 0.05)

As outlined in “Introduction”, we ran one-tailed paired *t* tests to see if ABA trial sequences had worse performance than CBA trial sequences when trial *n* − 1 was completed and when trial *n* − 1 was no-execution, to find out if each type of previous trial completion causes significant backward inhibition in and of themselves. If response execution is necessary for backward inhibition to be triggered, we would expect to see no *n* − 2 repetition cost following no-execution trials. Additionally, a one-tailed paired *t* test was run to examine whether the *n* − 2 repetition cost following completed trials was larger than the *n* − 2 repetition cost following no-execution trials. Finally, an exploratory a two-way repeated-measures ANOVA was run with two within-subject factors, trial sequence (ABA, CBA) and trial *n* − 1 completion (completed, no-execution). See Fig. 2 and Table 1 for summary data.

**Table 1:** Means (*M*) and standard deviations (SD) for RTs and error percentages on ABA and CBA trial sequences in Experiments 1, 2 and 3 by trial *n* − 1 completion

Experiment	Trial *n − *1 completion		ABA		CBA	
			M	SD	M	SD
*RT (ms)*						
Experiment 1	Completed		1193	226	1186	209
	No-execution		1210	199	1177	202
Experiment 2	Completed		874	100	841	125
	Cue-only		701	111	726	126
Experiment 3	Completed		1179	282	1116	283
	Cue-only		820	187	897	248
*Error (%)*						
Experiment 1	Completed		5.70	4.97	6.15	5.62
	No-execution		6.73	6.13	6.66	5.71
Experiment 2	Completed		7.67	4.51	7.43	4.53
	Cue-only		6.61	6.75	7.59	7.28
Experiment 3	Completed		9.43	6.69	8.71	6.17
	Cue-only		6.92	5.84	6.76	5.26

#### Reaction time

The *n* − 2 repetition cost following no-execution trials (33 ms) was significant, *t*(39) = 2.26, *p*_(one-tailed)_ = 0.015, *d*_z_ = 0.36, indicating that response execution is not necessary to trigger backward inhibition. Unexpectedly, the *n* − 2 repetition cost following completed trials (7 ms) was not significant, *t*(39) = 0.71, *p*_(one-tailed)_ = 0.242, *d*_z_ = 0.11. The *n* − 2 repetition cost following completed trials was not significantly larger than that following no-execution trials (a mean difference of − 26 ms), *t*(39) = − 1.45, *p*_(one-tailed)_ = 0.922, *d*_z_ = − 0.23, indicating that response execution did not increase the strength of inhibition triggered.

The main effect of trial sequence in the ANOVA was significant, *F*(1,39) = 5.59, MSE = 2826, *p* = 0.023, $\eta_{{\text{p}}}^{2}$ = 0.125, with ABA trial sequences producing slower responses (1201 ms) than CBA trial sequences (1181 ms). The main effect of trial *n* − 1 completion was not significant, *F*(1,39) = 0.09, MSE = 8238, *p* = 0.762, $\eta_{{\text{p}}}^{2}$ = 0.002 (completed: 1189 ms, no-execution: 1194 ms). The interaction between trial sequence and trial *n* − 1 completion was not significant, *F*(1,39) = 2.10, MSE = 3263, *p* = 0.156, $\eta_{{\text{p}}}^{2}$ = 0.051.

#### Percentage error

The *n* − 2 repetition cost following no-execution trials (0.07%) was not significant, *t*(39) = 0.09, *p*_(one-tailed)_ = 0.465, *d*_z_ = 0.014, and neither was the *n* − 2 repetition cost following completed trials (− 0.45%), *t*(39) = − 0.87, *p*_(one-tailed)_ = 0.806, *d*_z_ = − 0.138. The *n* − 2 repetition cost following completed trials was not significantly larger than that following no-execution trials (a mean difference of − 0.52%), *t*(39) = − 0.52, *p*_(one-tailed)_ = 0.697, *d*_z_ = − 0.082.

The main effect of trial sequence in the ANOVA was not significant, *F*(1,39) = 0.19, MSE = 7.70, *p* = 0.666, $\eta_{{\text{p}}}^{2}$ = 0.005 (ABA: 6.21%; CBA: 6.40%). The main effect of trial *n* − 1 completion was not significant, *F*(1,39) = 1.41, MSE = 16.77, *p* = 0.242, $\eta_{{\text{p}}}^{2}$ = 0.035 (completed: 5.92%, no-execution: 6.69%). The interaction between trial sequence and trial *n* − 1 completion was not significant, *F*(1,39) = 0.27, MSE = 9.98, *p* = 0.605, $\eta_{{\text{p}}}^{2}$ = 0.007.

### Discussion

Experiment 1 examined whether response execution is necessary to trigger backward inhibition by comparing completed and no-execution trials (where response execution was excluded). The results showed significant backward inhibition after no-execution trials, suggesting that response execution is not required. This result contradicts Philipp et al.’s (2007) finding that response execution on the previous trial was crucial for the occurrence of the *n* − 2 repetition cost, but it is consistent with studies indicating a key role for cue-related processing in the existence and size of the effect (Arbuthnott, 2005; Arbuthnott & Woodward, 2002; Astle, et al., 2012; Costa & Friedrich, 2012; Gade & Koch, 2014; Houghton et al., 2009; Hübner et al., 2003; Kuhns et al., 2007; Mayr & Keele, 2000; Prosser et al., 2020; Scheil & Kleinsorge, 2014a). Based on the results of Lenartowicz et al. we had suspected that the nogo signal could interfere with the effects of task processing, and so we removed the nogo element and used no-execution trials instead. Our finding of a significant a *n* − 2 repetition cost after no-execution trials implies that the lack of *n* − 2 repetition cost after nogo trials in Philipp et al.’s study could indeed have been due to interference from the nogo signal. The absence of backward inhibition following completed trials was an unexpected result for which we have no obvious explanation. It may be a Type 2 error, resulting from insufficient experimental power in our design to detect a significant cost following completed trials. We note that the exploratory ANOVA showed an overall significant main effect of trial sequence in the direction of an *n* − 2 repetition cost and no significant interaction with preceding trial completion, although the absence of an interaction might of course also result from there being insufficient power to detect one. It is possible that the substantial length of time (900 ms) between response on trial *n* − 1 and task cue presentation on trial *n* contributed to the measure of backward inhibition following completed trials being unreliable in Experiment 1; Scheil and Kleinsorge (2014b) found that the size of backward inhibition decreased at response–cue intervals over 300 ms. In the subsequent experiments, we changed the design slightly to encourage a more reliable backward inhibition effect.

It is possible that the *n* − 2 cost detected after no-execution trials in this experiment reflects to some extent the effect of episodic mismatch on ABA trials, whereby encountering the same task but with different trial details (target stimulus and/or response) creates interference and thereby slows responding. Because we followed Philipp et al.’s (2007) design for this experiment and ensured that the target stimulus changed every time the task re-occurred, there were no “match” trials against we could assess the effect of mismatch. Therefore, it is not possible to assess the extent to which interference from episodic mismatch might have contributed to the *n* − 2 cost measured here. (We note that in subsequent experiments there was no such restriction imposed upon on targets with re-occurring tasks.)

Having found that response execution is not required for backward inhibition, we went on to investigate what is required to trigger the effect. On the no-execution trials of Experiment 1, following which we did find a significant *n* − 2 repetition cost, participants had been able to prepare the cued task, process a target stimulus according to the cued task and select the appropriate response. Logically therefore, backward inhibition could have been triggered at (or prior to) any of those stages. To draw their conclusion that task preparation did not trigger backward inhibition, Schuch and Koch (2003) used the nogo method that Lenartowicz et al. (2011) have since found can prevent switch costs driven by task preparation from being seen on the following trial. Therefore, we chose to re-examine the question of whether task preparation could trigger backward inhibition, using a truncated-trial design. To do this, we simply truncated trials at an earlier stage of the trial than in Experiment 1 − i.e. after task preparation and before target onset. Since no target would be shown on these trials, the task itself would not be able to be performed at all. This allowed us to look for evidence of backward inhibition being triggered by task preparation.

## Experiment 2

This experiment examined whether backward inhibition can be triggered by task preparation using cue-only trials, as in Lenartowicz et al. (2011). Unlike no-execution trials in Experiment 1, no target was presented on cue-only trials. Therefore, target processing and response selection were excluded without any potential interference by a nogo signal. If the preparation stage triggers backward inhibition, then we would expect to see an *n* − 2 repetition cost following cue-only trials.

Furthermore, if preparation is the only source of backward inhibition within a trial, then we would expect no difference in the size of the *n* − 2 repetition cost after completed and cue-only trials. Alternatively, if backward inhibition is triggered both by preparation and target/response processing then other stages (post the cue stage) should increase the size of the *n* − 2 repetition cost already present following preparation. In contrast, if backward inhibition is only ever triggered by target/response processing, then the preparation stage will not trigger it and we would expect to see an *n* − 2 repetition cost only following completed trials.

To encourage a more reliable backward inhibition effect than that in Experiment 1 (which did not reach significance following completed trials), we replaced the number-judgement tasks with three tasks based on judgements of coloured shapes that have repeatedly produced *n* − 2 repetition costs in our lab and which would increase our confidence in producing *n* − 2 repetition costs again (Prosser, 2018; Prosser et al., 2020). We also reduced the post-response delay from 900 to 50 ms in this experiment.

### Methods

#### Participants

Forty participants were tested in total for course credit. One participant was excluded for having an accuracy rate of less than 70% and six participants were excluded for having more than 10% of response times faster than 200 ms or slower than 2000 ms. The upper bound was decreased from that used in Experiment 1 due to easier tasks being used. Of the 33 remaining participants 24 were female and their age ranged from 18 to 33 years old (mean age: 20.73 years).

#### Apparatus and stimuli

For this experiment and all following experiments, we used a colour-judgement task (blue and green for left response; red and yellow for right response), a shape-judgement task (square and triangle for left response; circle and diamond for right response) and a line-orientation-judgement task (slanted left and vertical for left response; slanted right and horizontal for right response). The task cue words were COLOUR, SHAPE, or LINE, shown in the centre of the screen. The targets were trivalent, consisting of coloured shapes that contained two lines in one of the four orientations. All stimulus combinations were possible and the combination of the colours, shapes and line orientation was completely random and therefore stimulus repetitions were allowed. The index finger of the dominant hand was used for all responses. It was rested on the middle of the three buttons between trials and moved left and right to the outer buttons to respond to targets. Task repetitions were not allowed. Roughly 28% of all experimental trials were cue-only trials, and cue-only trials could only occur after a completed trial.

#### Procedure

All the instructions were given on the screen. Participants initially started with a practice block of each task separately which each lasted for 20 trials. They then completed a practice block of 20 trials where they switched between the three tasks. The final practice block replicated an experimental block, in that participants completed 50 trials that involved switching between the three tasks and included cue-only trials.

Participants then completed the experimental blocks, of which there were 15 containing 50 trials each. After each block they were given the option to take a break and were given feedback of their average reaction time and total accuracy for that block to try to encourage better performance.

A trial began with a task cue being presented for 1000 ms, as seen in Fig. 1. On completed trials the task cue was then replaced by the target. The target stayed on the screen until participants gave a response. If participants gave a wrong response, feedback was given for 500 ms which said “WRONG BUTTON”. After the response/feedback on completed trials a blank screen was displayed for 50 ms, and then the next trial started. On cue-only trials the task cue was replaced by a blank screen for 50 ms. After the blank screen the next trial was started.

### Results

#### Data processing and analysis plan

The data were analysed essentially in the same manner as in Experiment 1. The planned analysis involved trial sequences where trials *n* and *n* − 2 were completed trials and trial *n − *1 was either a completed trial or a cue-only trial. The criteria for trial exclusion were the same as Experiment 1 except that the upper limit for trial exclusions based on reaction times was changed from 2500 to 2000 ms; 2.35% of all completed trials were excluded for having RTs below 200 ms or above 2000 ms. The percentage of trials with an inaccurate response on the current completed trial was 8.38%. For the RT analysis, after exclusions, there were on average 87 trials per trial sequence (ABA, CBA) per participant for previous cue-only trial sequences and on average 71 trials for previous completed trial sequences.

As with Experiment 1 we ran one-tailed paired *t* tests to see if ABA trial sequences had worse performance than CBA trial sequences when trial *n* − 1 was cue-only and when trial *n* − 1 was completed, to find out if each type of previous trial completion causes significant backward inhibition. If preparation is enough for backward inhibition to be triggered, we would expect an *n* − 2 repetition cost following cue-only trials (as well as following completed trials). Additionally, a one-tailed paired *t* test was run to examine whether the *n* − 2 repetition cost following completed trials was larger than the *n* − 2 repetition cost following cue-only trials. Finally, an exploratory two-way repeated-measures ANOVA was run with two within-subject factors, trial sequence (ABA, CBA) and trial *n* − 1 completion (completed, cue-only). See Fig. 2 and Table 1 for summary data.

#### Reaction time

There was no significant *n* − 2 repetition cost following cue-only trials (− 25 ms; note this is a relative benefit, rather than a cost, of *n* − 2 repetition), *t*(32) = − 2.27, *p*_(one-tailed)_ = 0.985, *d*_z_ = − 0.40, indicating that task preparation was not enough to trigger backward inhibition. There was a significant *n* − 2 repetition cost following completed trials, *t*(32) = 3.43, *p*_(one-tailed)_ < 0.001, *d*_z_ = 0.60, with ABA trial sequences being 33 ms slower than CBA trial sequences, indicating that completing all stages of task processing was enough to trigger backward inhibition. The *n* − 2 repetition cost following completed trials was significantly larger (a mean difference of 58 ms) than that following cue-only trials, *t*(32) = 5.36, *p*_(one-tailed)_ < 0.001, *d*_z_ = 0.93.

The main effect of trial sequence in the ANOVA was not significant, *F*(1,32) = 0.24, MSE = 2538, *p* = 0.627, $\eta_{{\text{p}}}^{2}$ = 0.007 (ABA: 787 ms; CBA: 783 ms). The main effect of trial *n* − 1 completion was significant, *F*(1,32) = 144.47, MSE = 4742, *p* < 0.001, $\eta_{{\text{p}}}^{2}$ = 0.819, with participants responding 144 ms slower following a completed trial (857 ms) than a cue-only trial (713 ms). The interaction between trial sequence and trial *n* − 1 completion was significant, *F*(1,32) = 28.76, MSE = 963, *p* < 0.001, $\eta_{{\text{p}}}^{2}$ = 0.473. Simple main effects analysis of this interaction using two-tailed *t* tests showed that effect of trial sequence following completed trials (an *n* − 2 repetition cost of 33 ms, reported above with a one-tailed test) was again significant, *p*_(two-tailed)_ = 0.002; however, following cue-only trials, the effect of trial sequence was in the opposite direction, with a 25 ms benefit (rather than a cost) of *n* − 2 repetition being significant with the two-tailed test, *p*_(two-tailed)_ = 0.030.

#### Percentage error

The *n* − 2 repetition cost following cue-only trials (− 0.99%) was not significant, *t*(32) = − 1.72, *p*_(one-tailed)_ = 0.953, *d*_z_ = − 0.30. The *n* − 2 repetition cost following completed trials (0.25%) was not significant, *t*(32) = 0.35, *p*_(one-tailed)_ = 0.366, *d*_z_ = 0.06. The *n* − 2 repetition cost following completed trials was not significantly larger than that following cue-only trials (a mean difference of 1.23%), *t*(32) = 1.45, *p*_(one-tailed)_ = 0.079, *d*_z_ = 0.25.

The main effect of trial sequence in the ANOVA was not significant, *F*(1,32) = 0.57, MSE = 7.85, *p* = 0.455, $\eta_{{\text{p}}}^{2}$ = 0.018 (ABA:7.14%, CBA: 7.51%). The main effect of trial *n* − 1 completion was not significant, *F*(1,32) = 0.20, MSE = 32.85, *p* = 0.656, $\eta_{{\text{p}}}^{2}$ = 0.006 (completed: 7.55%, cue-only: 7.10%). The interaction between trial sequence and trial *n* − 1 completion was not significant, *F*(1,32) = 2.09, MSE = 5.98, *p* = 0.158, $\eta_{{\text{p}}}^{2}$ = 0.061.

### Discussion

Experiment 2 investigated whether the preparation stage is sufficient to trigger backward inhibition. When trial *n* − 1 was completed, there was a significant *n* − 2 repetition cost. However, when trial *n* − 1 was a cue-only trial, there was no significant *n* − 2 repetition cost, and in fact the exploratory analysis found the effect was significant in the opposite direction, that is, there was a significant *n* − 2 repetition benefit. The *n* − 2 repetition benefit is likely caused by the task completed on trial *n* − 2 still being active when the participant performs the task on trial *n* in an ABA sequence. This implies that no inhibition of that task had been applied during the (*n* − 1) cue-only trials. Hence, it appears that the preparation stage did not trigger backward inhibition in this experiment.

Because task cues were removed from the screen when targets were presented, it seems likely that participants would have used them to some extent in advance of target onset, although we cannot be sure whether this would be simply to register the cue identity or to fully prepare to perform the task itself (e.g. by readying the appropriate target feature-response mappings). What is clear is that whatever processes did take place prior to target onset, they were insufficient to drive any detectable *n* − 2 repetition cost on the next trial. In contrast, completing the task (on completed trials) clearly was sufficient to generate the cost. Putting these results together with those from Experiment 1, it seems that the key stage occurred after target onset but before response execution, consistent with the proposal that response selection is responsible for driving the effect (Schuch & Koch, 2003). However, the absence of an *n* − 2 repetition cost following cue-only trials in this experiment does not necessarily mean that task backward inhibition could not be driven proactively in other circumstances, for instance where the need for proactive control was higher than it was here, such as where task cues did not directly indicate a specific task. Studies manipulating the transparency of mapping from cue to task have shown that backward inhibition was greater for less transparent task cues (Arbuthnott, 2005; Arbuthnott & Woodward, 2002; Gade & Koch, 2014; Houghton et al., 2009). From a proactive control point of view, this is important because active goal maintenance, along with goal activation, is a key element of proactive control (Braver, 2012) and if task cues allow for easy goal (task) activation, the need for goal maintenance might be low, as the goal can easily be reactivated when needed. On the other hand, if the activation of the goal requires more effort, then maintaining activation might be relatively more beneficial than when goal activation does not require much effort. The task cues used in the present experiment named the task itself (e.g. “COLOUR”), which might have been so easy to translate into a task goal that there was no need to actively maintain that task as a goal (Yamaguchi et al., 2021). If there was little need for the goal maintenance aspect of proactive control in this experiment, there might also have been little opportunity for any associated proactive backward inhibition to have been triggered. Therefore, in Experiment 3, we used arbitrary task cues to indicate the three tasks, to examine whether the preparation stage of task processing now triggered backward inhibition when the need for goal maintenance was increased.

## Experiment 3

This experiment was the same as Experiment 2 apart from the task cues were no longer the name of the task and instead they were "*****" for the colour task, "@@@@@" for the line-orientation task and "&&&&&" for the shape task.

### Methods

#### Participants

Forty-eight participants were tested. Three participants were excluded for having an accuracy rate of less than 70%, and none were excluded for having more than 10% of response times faster than 200 ms or slower than 4000 ms.^2^ Of the 45 remaining participants 27 were female and their age ranged from 17 to 37 years old (mean age: 22.1 years).

#### Apparatus and stimuli

This experiment was the same as Experiment 2 apart from the task cues were no longer the name of the task and instead they were “*****” for the colour task, “@@@@@” for the line-orientation task and “&&&&&” for the shape task. Additionally, due to software capabilities the response-to-cue interval was increased from 50 to 100 ms to allow for end of trial processing, and we removed the 50 ms blank following task cue presentation on cue-only trials.

### Results

#### Data processing and analysis plan

We analysed the mean RT for each participant and mean percentage of trials on which an error was made. The planned analysis involved trial sequences where trials *n* and *n* − 2 were completed trials and trial *n − *1 was either a completed trial or a cue-only trial. The criteria for trial exclusion were the same as Experiment 1 except that the upper limit for trial exclusions based on reaction times was changed from 2500 to 4000 ms (1.79% of all completed trials were outside of 200 and 4000 ms). The percentage of trials with an inaccurate response on the current completed trial was 9.66%. For the RT analysis, after exclusions there were on average 87 trials per trial sequence (ABA, CBA) per participant for previous cue-only trial sequences and on average 67 trials for previous completed trial sequences.

For this experiment and the following experiment, unlike the previous two experiments, we preregistered our plan for data processing and statistical analysis (see “Data availability” ). The analysis plan outlined in the introduction, and followed below, involves one deviation from the preregistration. We had preregistered a two-tailed (rather than one-tailed) *t* test to compare ABA trial sequence to CBA sequences following cue-only trials because we had wished to be able to test for the presence of a significant *n* − 2 repetition benefit (as found in Expt. 2) as well as testing for a potential *n* − 2 repetition cost. In hindsight, we realise that this would not allow us to consistently prioritise the central research question regarding backward inhibition (which would only be indicated by a cost, and not by a benefit, of *n* − 2 task repetition), so we report the results for a one-tailed test (rather than the preregistered two-tailed test) for an *n* − 2 repetition cost following cue-only trials as we have done for the other experiments in this paper. An exploratory (and not preregistered) two-way repeated-measures ANOVA was also run, with two within-subject factors, trial sequence (ABA, CBA) and trial *n* − 1 completion (completed, cue-only). Please note that the two-tailed test for an effect of trial sequence following cue-only trials that had been preregistered is still reported here as it equates to the test for the simple main effect of trial sequence following cue-only trials stemming from the ANOVA. See Fig. 2 and Table 1 for summary data.

#### Reaction time

Following cue-only trials, there was a not a significant *n* − 2 repetition cost (− 77 ms, note this is an *n* − 2 repetition benefit, rather than an *n* − 2 repetition cost), *t*(44) = − 5.01, *p*_(one-tailed)_ > 0.999, *d*_z_ = − 0.75, indicating that task preparation was not enough to trigger backward inhibition. Following completed trials, there was a significant *n* − 2 repetition cost (63 ms), *t*(44) = 3.76, *p*_(one-tailed)_ < 0.001, *d*_z_ = 0.56, indicating that that completing all stages of task processing was enough to trigger backward inhibition. The *n* − 2 repetition cost following completed trials was significantly larger (a mean difference of 140 ms) than that following cue-only trials, *t*(44) = 6.32, *p*_(one-tailed)_ < 0.001, *d*_z_ = 0.94.

The main effect of trial sequence in the ANOVA was not significant, *F*(1,44) = 0.38, MSE = 6112, *p* = 0.539, $\eta_{{\text{p}}}^{2}$ = 0.009 (ABA: 999 ms; CBA: 1006 ms). The main effect of trial *n* − 1 completion was significant, *F*(1,44) = 317.05, MSE = 11,863, *p* < 0.001, $\eta_{{\text{p}}}^{2}$ = 0.878, with participants responding 289 ms slower following a completed trial (1147 ms) than a cue-only trial (858 ms). The interaction between trial sequence and trial *n* − 1 completion was significant, *F*(1,44) = 39.99, MSE = 5514, *p* < 0.001, $\eta_{{\text{p}}}^{2}$ = 0.476. Simple main effects analysis using two-tailed *t* tests showed that the trial-sequence effect following completed trials (an *n* − 2 repetition cost of 63 ms, reported above with a one-tailed test) remained significant, *p*_(two-tailed)_ < 0.001, and that (as in Experiment 2) the trial-sequence effect was in the opposite direction following cue-only trials, an *n* − 2 repetition benefit of 77 ms being significant with the two-tailed test, *p*_(two-tailed)_ < 0.001.

### Percentage error

There was no significant *n* − 2 repetition cost following cue-only trials (0.17%), *t*(44) = 0.28, *p*_(one-tailed)_ = 0.390, *d*_z_ = 0.04. There was no significant *n* − 2 repetition cost following completed trials (0.73%), *t*(44) = 1.06, *p*_(one-tailed)_ = 0.148, *d*_z_ = 0.16. The *n* − 2 repetition cost following completed trials was not significantly larger than that following cue-only trials (a mean difference of 0.56%), *t*(44) = 0.61, *p*_(one-tailed)_ = 0.272, *d*_z_ = 0.09.

The main effect of trial sequence in the ANOVA was not significant, *F*(1,44) = 0.98, MSE = 9.19, *p* = 0.329, $\eta_{{\text{p}}}^{2}$ = 0.022 (ABA: 8.18%, CBA: 7.73%). The main effect of trial *n* − 1 completion was significant, *F*(1,44) = 20.14, MSE = 11.10, *p* < 0.001, $\eta_{{\text{p}}}^{2}$ = 0.314, with participants responding incorrectly 2.23% more following a completed trial (9.07%) than a cue-only trial (6.84%). The interaction between trial sequence and trial *n* − 1 completion was not significant, *F*(1,44) = 0.373, MSE = 9.37, *p* = 0.544, $\eta_{{\text{p}}}^{2}$ = 0.008.

### Discussion

Despite the use of arbitrary task cues, the pattern of results in Experiment 3 replicated Experiment 2: there was a significant *n* − 2 repetition cost when trial *n* − 1 was completed, but not when trial *n* − 1 was a cue-only trial. In fact, the *n* − 2 repetition effect constituted a benefit following cue-only trials, which was statistically significant in the exploratory analysis. As mentioned in the discussion of Experiment 2, the significant *n* − 2 repetition benefit is likely caused by activation from the task on trial *n* − 2 remaining and not being dampened by inhibition applied on trial *n* − 1. This result speaks against our suggestion above that it might have been the absence of a need for active task maintenance during the preparation interval that led to no backward inhibition being evident following cue-only trials in Experiment 2. Instead, despite the use of arbitrary cues in Experiment 3, we saw again a benefit of *n* − 2 repetition rather than the cost that would have indicated backward inhibition. Thus far, therefore, the evidence from these studies points towards backward inhibition being generated solely via processes occurring after target onset but before response execution (consistent with response selection being the key stage involved; e.g. Schuch & Koch, 2003); these results give no indication that backward inhibition can be triggered in advance of target onset.

However, we do not wish to conclude on the basis of the experiments presented so far that task preparation is necessarily incapable of generating backward inhibition. While Experiments 2 and 3 encouraged task preparation prior to target onset, they did not ensure that it took place, and nor did they provide any evidence that participants actually used the task cue to prepare the appropriate task prior to target presentation. While participants needed to remember the task cue that was presented (as it was not on the screen when the target was presented), they did not need to process it in detail prior to the target being presented. If participants did not use the task cue to prepare in advance, then finding no backward inhibition following cue-only trials would not constitute evidence that preparation is insufficient to trigger backward inhibition.^3^ To ensure that cue processing had occurred in advance of target onset, Experiment 4 used a double-registration procedure (Arrington et al., 2007) that required participants to respond to the task cue and indicate which task had been cued on every trial before they responded to the target.

## Experiment 4

This final experiment used a double-registration paradigm (see Arrington et al., 2007; Regev & Meiran, 2017; Van Loy et al., 2010). In this paradigm, participants respond twice on each trial: firstly to indicate which task has been cued, and secondly to perform that task. Importantly, participants cannot progress onto target processing until they have processed the task cue and selected the correct cue response. This way, we could be sure that they had processed the task cue enough to indicate the task they were about to complete. While this does not ensure that all aspects of task preparation have taken place, it does at least ensure that an important aspect of preparation (i.e. task identification) took place.

It is also important to note that cue responses require both response selection and response execution; however, these are not stages of task performance as they take place before the presentation of a target stimulus. Hence, this experiment still retains a clear separation between task preparation and task performance, despite the introduction of cue responses. Therefore, it remains the case that response selection and response execution as elements of task performance can only take place on completed trials, as it was in Experiments 2 and 3.

In addition to the issue of whether preparation triggers backward inhibition, the present double-registration procedure also examined whether the inhibition that is triggered on the preceding trial affects cue responses or target responses on the current trial. Regev and Meiran (2017) used the double-registration paradigm (with only completed trials) and found significant *n* − 2 repetition costs at both cue response and target response. They interpreted those costs as evidence that backward inhibition affects both the preparation-driven retrieval of the task identity (i.e. the slowing of cue responses on ABA trial sequences resulting in an *n* − 2 repetition cost) and target-driven retrieval of task information (i.e. the slowing of target responses on ABA trial sequences resulting in an *n* − 2 repetition cost). Assuming that we replicate Regev and Meiran’s (2017) results of a significant an *n* − 2 repetition cost following completed trials at both cue and target responses, we can then investigate whether, if preparation triggers backward inhibition, it affects both preparation-driven retrieval (cue responses) and target-driven retrieval (target responses) or just preparation-driven retrieval (cue responses).

### Methods

#### Participants

Forty-nine participants were tested in total for course credit. Two participants were excluded for having an accuracy rate of less than 70% on cue responses, three participants were excluded for having an accuracy rate of less than 70% on target responses, and a further two participants were excluded for having more than 10% of trials excluded from analysis for response times faster than 200 ms or slower than 2000 ms on cue responses. No participants were excluded for having more than 10% of trials excluded from analysis for response times faster than 200 ms or slower than 3000 ms on target responses.^4^ Of the 42 remaining participants 38 were female and their age ranged from 18 to 40 years old (mean age: 21 years).

#### Apparatus and stimuli

This experiment was based on Experiment 2, the key difference being that in this experiment participants responded to the task cue as well as the target. The responses were made on MasterCooler keyboards (msec-accuracy responses). For the cue responses, participants used their left hand and pressed buttons S (ring finger, colour task), D (middle finger, shape task) and F (index finger, line task). For the target responses, participants used their right hand and pressed buttons J (index finger, left response) and K (middle finger, right response). Unlike previous experiments the background was dark grey (RGB colour 20, 20, 20) rather than black or white.

### Procedure

The experimental set-up was the same as Experiment 2 apart from the following changes. The final practice block and the experimental blocks contained 62 trials, rather than 50 trials. During the break between blocks, when participants were given their average RT and their accuracy, participants were given extra feedback during the break that stated, “Remember, please try to be fast AND accurate!” if they made more than nine errors in that block.

A trial began with a task cue being presented for 200 ms (as seen in Fig. 1). The screen then went blank (dark grey) until a response was detected. At that point, there was a 100 ms delay (to accommodate computer processing) until the next object was presented. On completed trials the target was then presented for 200 ms. Again, the screen was then blank until 100 ms after a response was detected, at which point the next trial started. On cue-only trials, after the 100 ms post-cue-response delay, the next trial started.

If participants gave a wrong response at either the task cue or the target, feedback in magenta was given for 500 ms that said, “WRONG RESPONSE”. This was followed by a 500 ms blank screen, after which the next trial started (this meant that if a wrong response was given at task cue no target was presented).

#### Design

There were 60 analysable trials within each block (plus two unanalysable trials at the beginning at the block), exactly 18 of those trials (30%) being cue-only, which resulted in 270 cue-only trials within the whole experiment. Cue-only trials were always followed by completed trials. In total across the session, exactly 135 trials were ABA trial sequences with trial *n* − 1 as cue-only trials, and 135 were CBA trial sequences with trial *n* − 1 as cue-only trials. Of all the completed trials, 50% were ABA and 50% were CBA.

### Results

#### Data processing and analysis plan

The plan for data processing (including criteria for data exclusion) and analysis for this experiment was preregistered (see “Data availability” section) and was carried out without deviation, as follows. We analysed the mean RT and PE for each participant. The first two trials of every block were excluded, and trial sequences were only included if trials *n* and *n* − 2 were completed trials. If either the cue response or target response of either of the previous two trials (*n* − 2 and *n* − 1) was inaccurate then that trial (*n*) was excluded. For analysis of the cue and target responses the trial was excluded if the response time for the task cue was below 200 ms or above 2000 ms (3.11% of all experimental trials) and for RT analysis the whole trial would be excluded if the cue response was inaccurate (6.12% of all experimental trials). For analysis of the target response, the trial was excluded if the response time for the current target response was below 200 ms or above 3000 ms (1.38% of all completed trials) and for the RT analysis the current trial would be excluded if the target response was inaccurate (9.77% of all completed trials). For the RT analysis at cue responses, there were on average 101 trials per trial sequence per participant on previous cue-only trial sequences, and 69 trials per trial sequence per participant on previous completed trial sequences, after exclusions. For the RT analysis at target responses, there were on average 92 trials per trial sequence per participant on previous cue-only trial sequences, and 63 trials per trial sequence per participant on previous completed trial sequences, after exclusions.

**Fig. 3:**
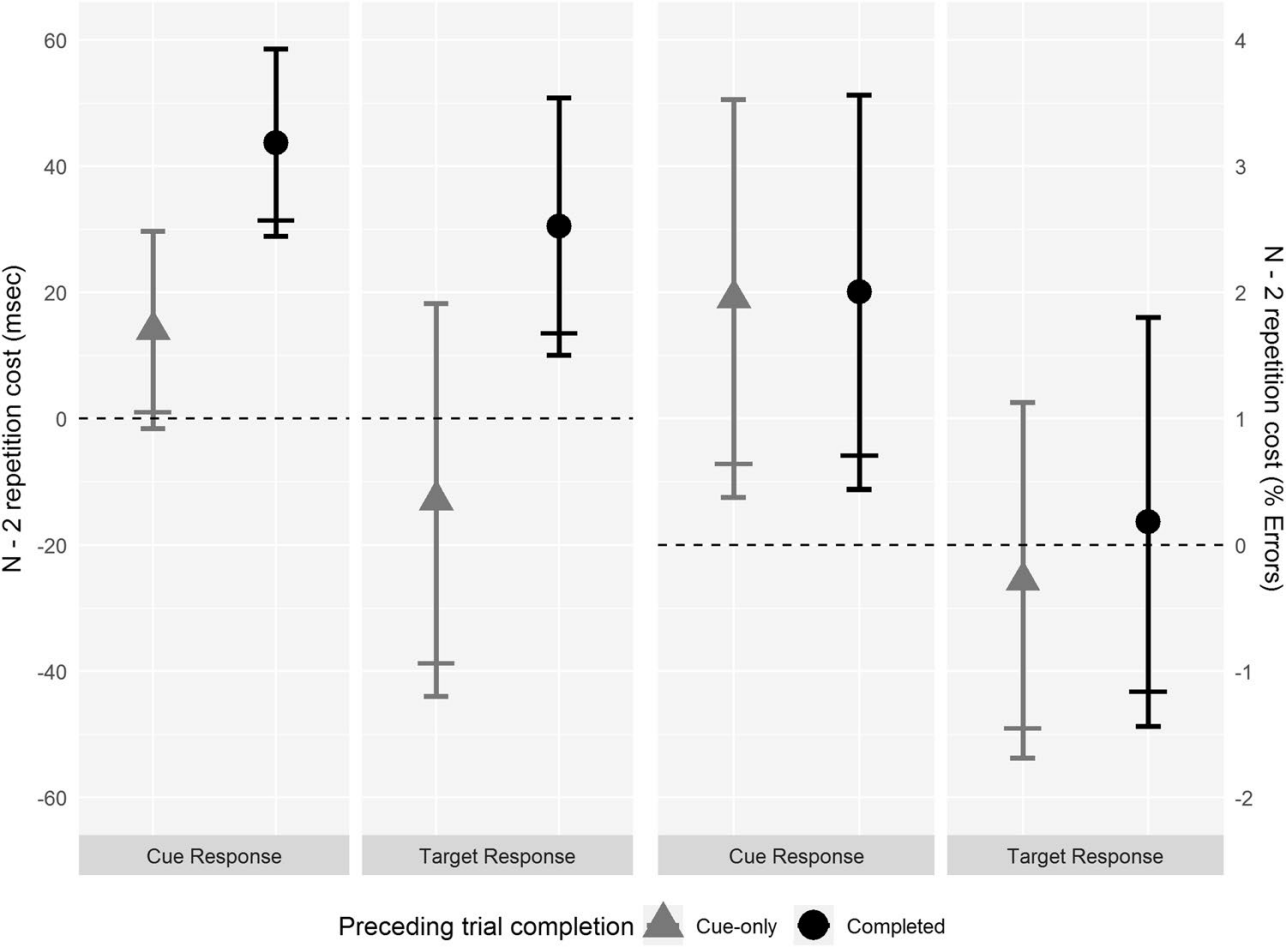
Experiment 4: *n* − 2 repetition cost for cue and target responses. Left side of the graph is RT data. Right side of the graphs is % error data. Grey triangles show data from completed trials following cue-only trials. Black circles show data from completed trials following completed trials. Narrow error bars above and below means show 95% CIs. Wide error bars below means show lower limit of 90% CI; where this is above the dashed zero line, the one-tailed *t* test for the presence of an *n* − 2 repetition cost is significant (*p* < 0.05)

Two one-tailed paired *t* tests were run to see if there was an *n* − 2 repetition cost following cue-only trials, one on cue-response data and one on target response data, both testing whether backward inhibition (affecting preparation and performance, respectively) could be triggered by preparation on the preceding trial. Two further planned one-tailed paired *t* tests were run to compare ABA and CBA trial sequences when trial *n* − 1 was completed, one on cue response data and one on target response data, testing whether we would replicate Regev and Meiran’s (2017) result that backward inhibition affects both cue processing and target processing. These four *t* tests, testing for the presence of an *n* − 2 repetition cost at cue and target responses following cue-only and completed trials, were all preregistered. See Fig. 3 and Table 2 for summary data.

**Table 2:** Means (*M*) and standard deviations (SD) of RTs and error percentages for cue and target responses on ABA and CBA trial sequences and mean and 95% confidence intervals (CI) for the *n* − 2 repetition cost by trial *n* − 1 completion for Experiment 4

Trial *n* − 1 completion	Cue responses				Target responses			
	ABA		CBA		ABA		CBA	
	M	SD	M	SD	M	SD	M	SD
RT (ms)								
Completed	797	114	753	103	1008	212	977	205
Cue-only	786	105	772	100	952	205	965	224
Error (%)								
Completed	6.42	4.96	4.42	3.97	8.93	5.13	8.74	5.72
Cue-only	6.85	5.50	4.90	4.19	8.20	5.58	8.48	5.30

We also ran two one-tailed paired *t* tests that were not part of the preregistration (but follow the plan stated in the introduction), that compared the *n* − 2 repetition cost following cue-only trials to the *n* − 2 repetition cost following completed trials, at cue responses and target responses. This analysis was to look at whether performance on the previous trial (i.e. completed trial) significantly increased the size of backward inhibition as compared to when only preparation could occur on the previous trial (i.e. cue-only trial). This analysis is followed by an exploratory (and not preregistered) two-way repeated-measures ANOVA with two within-subject factors, trial sequence (ABA, CBA) and trial *n* − 1 completion (completed, cue-only), run at each response level.

#### Reaction time

##### *Cue response*

There was a significant *n* − 2 repetition cost of 14 ms following cue-only trials for cue responses, *t*(41) = 1.82, *p*_(one-tailed)_ = 0.038, *d*_z_ = 0.28, indicating that completing task preparation was enough to trigger backward inhibition. There was also a significant *n* − 2 repetition cost of 44 ms following completed trials, *t*(41) = 5.95, *p*_(one-tailed)_ < 0.001, *d*_z_ = 0.92, indicating that completing all stages of task processing was enough to trigger backward inhibition. The *n* − 2 repetition cost following completed trials was significantly larger (a mean difference of 30 ms) than that following cue-only trials, *t*(41) = 3.30, *p*_(one-tailed)_ = 0.001, *d*_z_ = 0.51, indicating that stages after task preparation increased the strength of backward inhibition triggered.

Analysis of variance showed a significant main effect of trial sequence, *F*(1,41) = 22.66, MSE = 1550, *p* < 0.001, $\eta_{{\text{p}}}^{2}$ = 0.356, with participants responding on average 29 ms slower on ABA trial sequences (791 ms) than CBA trial sequences (762 ms). The main effect of trial *n* − 1 completion was not significant, *F*(1,41) = 0.15, MSE = 5605, *p* = 0.697, $\eta_{{\text{p}}}^{2}$ = 0.004, (completed trial: 775 ms, cue-only trial: 779 ms). The interaction between trial sequence and trial *n* − 1 completion was significant, *F*(1,41) = 10.89, MSE = 849, *p* = 0.002, $\eta_{{\text{p}}}^{2}$ = 0.210. The effect of trial sequence following completed trials (an *n* − 2 repetition cost of 44 ms, reported above with a one-tailed test) was significant when tested with a two-tailed *t* test, *p*_(two-tailed)_ < 0.001; the effect of trial sequence following cue-only trials (an *n* − 2 repetition cost of 14 ms), was not significant with a two-tailed test, *p*_(two-tailed)_ = 0.077.

##### *Target response*

There was no significant *n* − 2 repetition cost following cue-only trials (− 13 ms) for target responses, *t*(41) = − 0.83, *p*_(one-tailed)_ = 0.795, *d*_z_ = − 0.13, indicating that task preparation was not enough to trigger backward inhibition. There was a significant *n* − 2 repetition cost following completed trials (31 ms), *t*(41) = 3.02, *p*_(one-tailed)_ = 0.002, *d*_z_ = 0.47, indicating that completing all the stages of task processing was enough to trigger backward inhibition. The *n* − 2 repetition cost following completed trials was significantly larger (43 ms) than that following cue-only trials, *t*(41) = 2.73, *p*_(one-tailed)_ = 0.005, *d*_z_ = 0.42.

The main effect of trial sequence in the ANOVA was not significant, *F*(1,41) = 0.73, MSE = 4485, *p* = 0.397, $\eta_{{\text{p}}}^{2}$ = 0.018, (ABA: 980 ms, CBA: 971 ms). The main effect of trial *n* − 1 completion was significant, *F*(1,41) = 4.40, MSE = 10,859, *p* = 0.042, $\eta_{{\text{p}}}^{2}$ = 0.097, with responses being 34 ms slower following completed trials (993 ms) as compared to following cue-only trials (959 ms). The interaction between trial sequence and trial *n* − 1 completion at target response was significant, *F*(1,41) = 7.48, MSE = 2640, *p* = 0.009, $\eta_{{\text{p}}}^{2}$ = 0.154. Simple main effects analysis of the trial-sequence effect showed that the 31 ms *n* − 2 repetition cost following completed trials (reported above with a one-tailed test) remained significant with the two-tailed test, *p*_(two-tailed)_ = 0.004, whereas there was no significant effect of trial sequence following cue-only trials, the numerical *n* − 2 repetition benefit of 13 ms not being statistically significant, *p*_(two-tailed)_ = 0.409.

#### Percentage error

##### *Cue response*

There was a significant *n* − 2 repetition cost following cue-only trials in the error data (1.96%), *t*(41) = 2.51, *p*_(one-tailed)_ = 0.008, *d*_z_ = 0.39; this is consistent with the significant cost in the RT data, indicating that task preparation is sufficient to trigger backward inhibition affecting cue responses. There was a significant *n* − 2 repetition cost following completed trials (2.01%), *t*(41) = 2.60, *p*_(one-tailed)_ = 0.007, *d*_z_ = 0.40. There was no significant increase in *n* − 2 repetition cost following completed trials as compared to that following cue-only trials (a mean difference of 0.05%), *t*(41) = 0.04, *p*_(one-tailed)_ = 0.482, *d*_z_ = 0.01.

The main effect of trial sequence in the ANOVA was significant, *F*(1,41) = 12.78, MSE = 12.89, *p* < 0.001, $\eta_{{\text{p}}}^{2}$ = 0.238, with participants making 1.98% more errors on ABA trial sequences (6.64%) than CBA trial sequences (4.66%). The main effect of trial *n* − 1 completion was not significant, *F*(1,41) = 0.77, MSE = 11.13, *p* = 0.385, $\eta_{{\text{p}}}^{2}$ = 0.018, (completed trial: 5.42%, cue-only trial: 5.87%). The interaction between trial sequence and trial *n* − 1 completion was not significant, *F*(1,41) = 0.002, MSE = 12.44, *p* = 0.965, $\eta_{{\text{p}}}^{2}$ < 0.001.

##### Target response

There was no significant *n* − 2 repetition cost following cue-only trials (− 0.28%), *t*(41) = − 0.40, *p*_(one-tailed)_ = 0.654, *d*_z_ = − 0.06. There was no significant *n* − 2 repetition cost following completed trials (0.19%), *t*(41) = 0.23, *p*_(one-tailed)_ = 0.409, *d*_z_ = 0.04. There was no significant increase of *n* − 2 repetition cost following completed trials as compared to that following cue-only trials (a mean difference of 0.47%), *t*(41) = 0.48, *p*_(one-tailed)_ = 0.316, *d*_z_ = 0.07.

The main effect of trial sequence in the ANOVA was not significant, *F*(1,41) = 0.01, MSE = 13.91, *p* = 0.936, $\eta_{{\text{p}}}^{2}$ < 0.001, (ABA: 8.56%, CBA: 8.61%). The main effect of trial *n* − 1 completion was not significant, *F*(1,41) = 1.31, MSE = 7.90, *p* = 0.259, $\eta_{{\text{p}}}^{2}$ = 0.031 (completed trial: 8.84%, cue-only trial: 8.34%). The interaction between trial sequence and trial *n* − 1 completion was not significant, *F*(1,41) = 0.23, MSE = 9.77, *p* = 0.632, $\eta_{{\text{p}}}^{2}$ = 0.006.

### Discussion

Unlike the preceding experiments, this experiment required responses to be made to task cues as well as to targets and therefore allowed us to demonstrate that participants prepared (at least to the extent of identification) the cued task on cue-only trials. Following cue-only trials, there was a significant *n* − 2 repetition cost at cue responses in terms of both RT and errors. The RT cost was small in terms of absolute size (14 ms) and, while it was statistically significant using the one-tailed test (as preregistered), we note that it would not have been significant had we used a two-tailed test. We consider the one-tailed test to have been appropriate since we were looking specifically for evidence of backward inhibition and therefore a cost (rather than simply an effect) of trial sequence, and the one-tailed test offers greater statistical power (and protection against a Type 2 error) for that specific prediction. Nevertheless, as with any significant result it could of course represent a Type 1 error and conclusions based upon this result alone should be considered somewhat tentative for the time being.

The finding of a significant *n* − 2 repetition cost following cue-only trials suggests that preparation can trigger backward inhibition. The fact that the cost occurred at cue responses indicates that the backward inhibition triggered by task preparation had the effect of slowing the process of identification of the task that had been switched away from previously. While this effect must have been triggered during the preparation part of the preceding trial (since no target is presented on cue-only trials), it would not constitute proactive control in the sense of acting in anticipation of, and to diminish, conflict expected to occur during future task performance. Rather, it seems likely to represent the persistence of inhibition generated to deal with conflict occurring at the time of task preparation, between alternative potential task-identity judgements. Nevertheless, it might well contribute to the standard backward inhibition effect measured in paradigms where only one response (to the target) is required per trial, especially if preparation times are short such that a slowing of task identification will impact upon the overall RT. It could be argued that there might have been no conflict at the task-identification stage if there had not been the requirement to signal the result of target identification via a specific overt response; in other words, the *n* − 2 repetition cost in cue responses might be an artefact resulting from using the double-registration procedure. This is an open question that would need to be addressed in future experiments. Notably, unlike the cost seen in Experiment 1 following no-execution trials (truncated after response selection), the cost seen here following cue-only trials (truncated after cue responses) could not result from an episodic mismatch effect, because all cue stimuli and cue responses for a particular task would repeat every time that task was used (i.e. there was no “mismatch” in this case that could cause interference). Hence, the *n* − 2 repetition cost does seem to represent inhibition in this case.

In Experiments 2 and 3, we found no evidence of backward inhibition following cue-only trials, contrasting with the finding of backward inhibition in cue responses following cue-only trials in Experiment 4. A number of reasons might account for that discrepancy. First, in Experiments 2 and 3 we did not ensure that task preparation took place on cue-only trials; the absence of any subsequent backward inhibition might therefore simply result from preparation not having occurred, rather than indicating that preparation did not drive backward inhibition. In Experiment 4, in contrast, we required responses to cues to identify the cued task, ensuring that at least an element of task preparation did take place. Second, in Experiments 2 and 3 RTs were measured from the time of onset of the target stimulus, but following a 1000 ms cue-target interval in each case. So if preparation on the preceding trial had triggered backward inhibition that slowed the process of cue-interpretation (as indicated by the *n* − 2 repetition cost following cue-only trials in Expt. 4), it is unlikely that such a small effect would have been observable in the RTs measured relative to onset of the target in those earlier experiments. In Experiment 4 there was no analogous time-period (prior to the onset of the event from which cue-RTs were measured) during which this cost could have been overcome prior to it being measured. Finally, as noted above, we cannot rule out the possibility that the backward inhibition observed in cue responses in Experiment 4 resulted solely from competition between overt responses rather than from task-identification processes.

While an *n* − 2 repetition cost following cue-only trials was only found at cue responses, following completed trials there was also a significant *n* − 2 repetition cost at target responses and the cost at cue responses was larger than that following cue-only trials. The difference in size of costs at cue responses following the two types of trial completion suggests that while preparation did trigger inhibition, completed trials apparently triggered stronger inhibition of the same processes (related to cue identification). The presence of a significant cost at target responses following completed but not cue-only trials might indicate that task performance triggers an additional element of inhibition, affecting the use of task information to make a target response that is not triggered by preparation alone. However, we might expect that with no inhibition at all we should see a significant *n* − 2 repetition benefit (as in Experiments 2 and 3), whereas no significant benefit was present at target responses following cue-only trials (although we note that there was a numerical benefit in both RTs and errors). The absence of either a significant *n* − 2 repetition cost or benefit might indicate that just enough inhibition was triggered to overcome the activation from the preceding trial, but not enough inhibition to be detrimental to performance on the subsequent trial (see Grange et al., 2013, for computational modelling evidence). Therefore, we refrain from concluding on the basis of these data that task preparation is insufficient to trigger backward inhibition that affects target responses. In other words, it is not clear from these data whether or not task preparation might be capable of triggering a “true” proactive-control effect that would prevent or offset anticipated conflict associated with future task performance.

Finally, we note that we replicated Regev and Meiran’s (2017) finding of backward inhibition affecting both preparation and performance of a task: following completed trials, there was a significant *n* − 2 repetition cost at cue responses and at target responses.

## General discussion

This set of experiments investigated which stage of task processing triggers the backward inhibition effect. We used a truncated-trial design to exclude stages of processing from taking place on trial *n* − 1: with this design, the presence of an *n* − 2 repetition cost following truncated trials shows that the excluded stages are not necessary to trigger backward inhibition. Experiment 1 truncated trials after response selection; an *n* − 2 repetition cost was observed after these no-execution trials, indicating that response execution is not necessary to trigger backward inhibition. Experiments 2 and 3 truncated trials after task preparation. Following these cue-only trials, there was a benefit rather than a cost of *n* − 2 repetition, and hence no evidence that the preparation stage is sufficient to trigger backward inhibition; however, the lack of confirmation that participants had prepared the task before target onset, together with the use of a long cue-target interval, prevented the strong conclusion that preparation could not trigger the effect. Experiment 4, in contrast, required participants to respond to the task cue as well as to the target stimulus, ensuring that they prepared the cued task on cue-only trials at least to the level that they could identify the relevant task. It also allowed us to examine backward inhibition at both the preparation stage and the performance stage of the current trial. Here, we found evidence of backward inhibition affecting preparation itself (in cue responses). This effect was small in terms of RT (14 ms, and not significant with the less powerful two-tailed *t* test) and therefore may be considered tentative at present, although we note that it was mirrored by a significant effect in errors. There was neither a cost nor a benefit of *n* − 2 task repetition following cue-only trials in target responses. Across Experiments 2–4, there was robust evidence that backward inhibition following completed trials, on which both task preparation and task performance took place, was stronger than any following cue-only trials, on which only task preparation took place.

The finding of backward inhibition after no-execution trials in Experiment 1 differs from that of Philipp et al. (2007), who found that response execution was necessary to trigger backward inhibition. It might be that their use of nogo trials rather than truncated trials had prevented the effects of response selection or earlier stages of task processing from being visible (c.f. Lenartowicz et al., 2011). Our results from Experiments 2 and 3, where we found no evidence of backward inhibition following cue-only trials, are consistent with those of Schuch and Koch (2003), who used a nogo design and found no backward inhibition following the preparation stage of task processing, but they are less consistent with studies showing an effect of task cue presence and type on the size of backward inhibition (Arbuthnott, 2005; Arbuthnott & Woodward, 2002; Gade & Koch, 2014; Houghton et al., 2009; Prosser et al., 2020). However, the finding in Experiment 4 of backward inhibition following cue-only trials is consistent with the implication of those studies that cue-related processing influences the size of the backward inhibition effect.

The idea that backward inhibition is closely tied to cue processing was first proposed by Mayr and Keele (2000) who initially proposed that backward inhibition could be a proactive control mechanism, and many studies have since shown cue-related effects on backward inhibition that make some role for cue processing in generating at least part of the effect seem likely (e.g. Arbuthnott, 2005; Arbuthnott & Woodward, 2002; Astle, et al., 2012; Costa & Friedrich, 2012; Gade & Koch, 2014; Hübner et al., 2003; Kuhns et al., 2007; Prosser et al., 2020; Scheil & Kleinsorge, 2014a). Houghton et al. (2009) argued that one target of backward inhibition is likely to be the cue-to-task translation process, whereby competition from the most recent translation (used on trial *n* − 2) competes (on trial *n* − 1) and is therefore suppressed, causing slowing of the same process on trial *n* of ABA sequences. The method used in Experiment 4 enabled us to test the implication of these studies—that backward inhibition may be triggered during cue processing—more directly than is possible when all trials are completed. The cue-only version of the truncated-trial method allows us to rule out all trial events that would usually occur after task preparation (target and response processing) as being the triggers of a subsequently observed effect, and the finding of an *n* − 2 repetition cost following cue-only trials is consistent with cue processing directly triggering a degree of backward inhibition, although we cannot at this stage distinguish between the roles of processing the cue itself and processing the cue response in generating the effect. The double-registration method allows us to determine whether inhibition impacts upon cue processing or target processing. Regev and Meiran (2017) used the method to show that both cue responses and target responses can show an *n* − 2 repetition cost following completed trials; we replicated that finding in Experiment 4, which combined the double-registration and truncated-trial techniques, and also showed that backward inhibition that was generated during the preparation stage of the preceding trial had its impact on the preparation stage of the current trial.

Backward inhibition has sometimes been posited as being triggered only in response to conflict that arises during performance (Gade & Koch, 2007; Koch, et al., 2010; Schuch & Koch, 2003). For instance, Koch et al.’s (2010) review on backward inhibition suggested that backward inhibition is a reactive control measure that is applied once conflict between tasks is detected, and this conflict arises during task performance, i.e. after cue processing. Correspondingly, a computational model of backward inhibition by Sexton and Cooper (2017) has backward inhibition being triggered in response to conflict during performance of the task, and does not include a mechanism whereby task preparation could cause backward inhibition. While the data from all of the experiments presented here clearly support a role for performance-related processes in driving a large part of the measured backward inhibition effect, the results from Experiment 4 suggest that there was also an element of backward inhibition that was triggered prior to task performance in that experiment. Nevertheless, it is important to note that we only found a clear cost of *n* − 2 task repetition following cue-only trials with respect to cue responses, whereby backward inhibition would be affecting task identification (or, potentially, selection of the responses used to indicate task identity) rather than task performance. The absence of neither a significant cost nor benefit of *n* − 2 task repetition in target responses following cue-only trials is an ambiguous result that is potentially consistent with a degree of backward inhibition (see Grange et al., 2013), so it remains to be seen whether future studies will find any evidence for an aspect of backward inhibition that affects task performance but that was triggered proactively, prior to target onset on the preceding trial.

In contrast to the tentative nature of the evidence for preparation-driven backward inhibition, we found evidence in all our experiments, including Experiment 4, that backward inhibition can be applied during task performance, and the relatively larger and more statistically robust costs following completed than cue-only trials seen in Experiments 2–4 suggests that backward inhibition generated during task performance may be much more substantial than any generated during task preparation. In Experiment 4, the *n* − 2 repetition cost at cue responses was increased, and an *n* − 2 repetition cost at target responses became evident, following task performance (i.e. following completed trials in comparison to cue-only trials), suggesting that more inhibition − and possibly inhibition of additional processes − was applied on completed trials in comparison with cue-only trials.

We note that it is possible that two issues might confound our interpretation that the difference in the *n* − 2 repetition cost on target responses following completed and cue-only trials was due to task performance occurring only on completed trials. First, completed trials were roughly 975 ms longer than cue-only trials. This increased length, rather than the addition of performance per se, might potentially have allowed more inhibition to be applied to the competing task during completed trials than during cue-only trials. Second, the degree of task preparation required to respond to a task cue might not be the same as that required to prepare fully to complete a task, and so it might be that more preparation occurred after the response to the task cue was given; if so, it might mean that more preparation occurred on completed trials than on cue-only trials (also see Swainson et al., 2021). This potential extra preparation might have caused the increase in backward inhibition measured at cue responses and target responses following completed trials.

Finally, as noted above, we reiterate that the *n* − 2 repetition cost is not necessarily an unambiguous sign of inhibition. It has been suggested to reflect the interfering effect of a mismatch between the current trial and the most recent instance of the same task in episodic memory when a task repeats with different trial features (i.e. target stimulus or response). Since such a mismatch effect could only occur with repetition of a recent task (e.g. ABA sequences), it can potentially inflate or even account for the *n* − 2 repetition cost (Gade et al., 2017; Grange et al., 2017; see also Mayr, 2002). It is possible that episodic mismatch could at least partly account for the *n* − 2 repetition cost that we measured for target responses in all the experiments reported here. Therefore, as noted in the “Discussion” section of Experiment 1, the *n* − 2 repetition cost that followed trials truncated after response-selection could potentially reflect interference rather than inhibition. However, it cannot account for the cost seen at cue responses in Experiment 4, including that following cue-only trials, since on ABA sequences the cue stimulus and cue response on trial *n* would always have matched those on trial *n* − 2.

## Conclusion

These experiments examined whether the backward inhibition effect was generated during, or prior to, task performance. We investigated which stages of task processing were responsible for triggering the effect, using a truncated-trial method. The results indicate that response execution is not necessary for backward inhibition to be triggered, and also that backward inhibition affecting task-identity responses on the current trial can be triggered by processes up to and including a task-identity response on the preceding trial. The results also indicate that when task performance occurs as well as task preparation, there is increased inhibition of cue processing as well as inhibition of task performance.

## Supplementary Information

Below is the link to the electronic supplementary material.

Supplementary file1 (PDF 187 KB)

## Data Availability

The data for Experiment 1 can be requested by emailing the corresponding author: r.swainson@abdn.ac.uk. The data for Experiment 2 and the experiment described in Online Resource 1 can be accessed at https://dx.doi.org/10.5255/UKDA-SN-855962. The data for Experiments 3 and 4 can be accessed at https://dx.doi.org/10.5255/UKDA-SN-854753. The preregistration for Experiment 3 can be found at https://aspredicted.org/jn74y.pdf and the preregistration for Experiment 4 at https://aspredicted.org/769eh.pdf.

## Footnotes

1 Our intention in Experiments 1, 2 and the supplementary experiment was to include a minimum of 30 participants’ data. Thirty participants would give us an 80% probability of detecting an effect size (*d*_z_) of 0.46 for a one-tailed paired *t* test (0.53 for two-tailed test), using an alpha of 0.05. Experiments 3 and 4 were run after Experiments 1, 2 and the supplementary experiment, and they were pre-registered. For these, the smallest sample size acceptable was increased to 40 which, given the same conditions above, would allow us to detect an effect size (*d*_z_) of 0.4 for a one-tailed test (0.45 or above for a two-tailed test). Power analyses used G*Power 3.1.9.4 (Faul et al., 2007).

2 As dictated by our preregistered analysis plan the speed restriction upper limit was increased from the 2000 ms, used in Experiment 2, to 4000 ms because more than 10% of the remaining participants were being excluded at the lower cut off. It is likely that participants responded more slowly in this experiment because of the use of arbitrary task cues.

3 In a separate experiment, we varied the cue-target interval with the aim of demonstrating that some preparation would have occurred on cue-only trials, but because of a possible confound between passive dissipation and active preparation, together with inconclusive results as to whether backward inhibition was triggered (there was neither a significant cost nor benefit of *n* − 2 repetition following cue-only trials), we do not report that study here (but see Online Resource 1 for full details, including “Methods” and “Results”).

4 Note the target response speed restriction was increased to 3000 ms from 2000 ms, as per preregistered analysis plan, since six participants would have been removed with the 2000 ms upper limit which was over 10% of the remaining participant sample.
