# Supplementary material for: Investigating task preparation and task performance as triggers of the backward inhibition effect
Source: Psychol Res. 2022 Dec 26;87(6):1816–35. doi: 10.1007/s00426-022-01780-x (PMC10366292; doi:10.1007/s00426-022-01780-x)
Supplement: Supplementary file 1 — Supplementary file1 (PDF 187 KB) [file 426_2022_1780_MOESM1_ESM.pdf]

Investigating task preparation and task performance as triggers of the backward inhibition effect

Psychological Research: Online Resource 1

Laura Joy Prosser[1], Motonori Yamaguchi[2], Rachel Swainson[1*]

[1] School of Psychology, University of Aberdeen
[2] Department of Psychology, University of Essex
* Corresponding author: r.swainson@abdn.ac.uk

Supplementary Experiment

Introduction

In Experiments 2 and 3 we attempted to test whether the preparation stage of task processing could be enough to trigger backward inhibition. However, we had no measure of task preparation, and so while we did not have any evidence of backward inhibition after cue-only trials we also had no evidence that any preparation occurred. Schuch and Koch (2003) were able to draw their interpretation that the cue stage was not enough to trigger backward inhibition because they varied the cue-target interval. They then compared reaction times between the two cue-target intervals (100ms vs 1000ms). They reported that there was a significant effect of cue-target interval on RT, with participants responding faster when the cue was on the screen for longer before target-onset. They interpreted this as showing that preparation occurred.

This experiment aimed to test whether the preparation stage of task processing could be enough to trigger backward inhibition, while also looking for evidence of preparation. We

included a 300ms cue-target interval, as well as the 1000ms interval used in Experiments 2 and 3, with the aim of using an improvement in performance with a long cue-target interval as evidence that preparation had taken place (although, as noted below, such improvement might actually reflect a passive effect of time since the preceding trial rather than an active effect of task preparation). If preparation is enough to trigger backward inhibition, we would expect that there would be a significant n – 2 repetition cost following cue-only trials.

Methods

Participants

Forty participants were tested in total for course credit. One participant was excluded for having an accuracy rate of less than 70% and four participants were excluded for having more than 10% of trials excluded from analysis for response times faster than 200ms or slower than 2000ms. Of the 35 remaining participants 30 were female and their age ranged from 18-27 years old (mean age: 20.05 years).

Materials

The tasks, cues and responses were the same as in Experiment 2.

Procedure

The procedure was the same as Experiment 2 apart from that the cue was presented for 300ms on 50% of completed trials and for 1000ms on the other 50%. In addition, to allow there to be enough trials for analysis the experimental blocks consisted of 75 trials (rather than 50) and there were now 25 blocks rather than 15.

Results

Data Processing and Analysis Plan

We analysed the mean reaction time (RT) for each subject and the mean percentage of trials on which an error was made (PE). For the analysis that included trial-sequences (ABA, CBA), the exclusions below were applied. The first two trials of every block were excluded, as were trials that had RTs below 200ms or above 2000ms (4% of all completed trials), and trial sequences were only included if trials *n* and *n* – 2 were completed trials. If the response of either of the previous two trials (*n* – 2 and *n* – 1) was inaccurate then that trial (*n*) was excluded. Additionally, for the RT analysis the current trial was excluded if the response was inaccurate (9.52% of all completed trials). Only trials where on trial *n* – 1 the cue-target interval (CTI) was 1000ms were included in analysis of trial sequences; CTI on trials *n* – 2 and *n* could be either 300ms or 1000ms. For the RT analysis, after exclusions there were on average 123 trials per trial sequence per participant on previous cue-only trial sequences, and 180 trials per trial sequence per participant on previous completed trial sequences.

To check if increasing cue-target interval improved performance, we ran a one-tailed *t* test comparing performance (RT and PE) between trials with 300ms and 1000ms CTI. For this analysis, we only included completed trials, we excluded trials if the RT was below 200ms or above 2000ms, and for the RT analysis the trial was excluded if the response was incorrect.

As per the main manuscript we ran three one-tailed *t* tests to answer our three key questions. The first question was whether the preparation stage of task processing is sufficient to trigger backward inhibition. The second question was whether backward inhibition is present following completed trials. The final question was whether the stages that had been excluded from occurring on the cue-only trials can increase the strength of the backward inhibition found when they occur on completed trials. Additionally, we ran an exploratory

two-way repeated-measures ANOVA with two within-subject factors, trial sequence (ABA, CBA) and trial *n* – 1 completion (completed, cue-only). See Supplementary Table for summary data.

Reaction Time

Reaction times were significantly shorter when there was a long cue-target interval (797ms) as compared to a short interval (904ms), $t(34) = 13.79$, $p_{(one\text{-}tailed)} < .001$, $d_z = 2.33$, indicating that the longer interval led to improved performance.

The *n* – 2 repetition cost following cue-only trials (0ms) was not significant, $t(34) = -0.07$, $p_{(one\text{-}tailed)} = .529$, $d_z = -0.013$, indicating that that task preparation was not enough to trigger backward inhibition. There was a significant *n* – 2 repetition cost following completed trials (16ms), $t(34) = 3.34$, $p_{(one\text{-}tailed)} = .001$, $d_z = 0.57$, indicating that completing all stages of task processing was enough to trigger backward inhibition. The *n* – 2 repetition cost was significantly larger (a mean difference of 16ms) following completed than cue-only trials, $t(34) = 1.97$, $p_{(one\text{-}tailed)} = .029$, $d_z = 0.33$.

The main effect of trial sequence in the ANOVA was not significant, $F(1,34) = 3.59$, MSE = 550, $p = .067$, $\eta_p^2 = .096$ (ABA: 827ms; CBA: 819 ms). The main effect of trial *n* – 1 completion was significant, $F(1,34) = 63.41$, MSE = 2490, $p < .001$, $\eta_p^2 = .651$, with participants responding 67ms slower following a completed trial (857ms) than a cue-only trial (790ms). The interaction between trial sequence and trial *n* – 1 completion was not significant, $F(1,34) = 3.86$, MSE = 581, $p = .058$, $\eta_p^2 = .102$.

Supplementary Table

Means (M) and standard deviations (SD) for RTs and error percentages on ABA and CBA trial sequences and mean and 95% confidence intervals (CI) for the n – 2 repetition cost in each experiment by trial n – 1 completion.

Experiment	Trial *n* – 1 completion	ABA		CBA		*n* – 2 repetition cost		
		M	SD	M	SD	M	95% CI [LL,UL]	90% CI LL
RT(ms)	Completed	865	135	849	130	16	[6.08, 24.95]	7.67
	Cue-only	789	129	790	133	0	[-13.82. 12.85]	-11.58
Error (%)	Completed	8.11	6.25	8.77	7.15	-0.66	[-1.75, 0.43]	-1.56
	Cue-only	8.26	7.28	9.42	9.23	-1.16	[-2.48, 0.16]	-2.26

Percentage Error

Participants made significantly fewer errors when there was a long cue-target interval (9.62%) as compared to a short interval (8.87%), $t(34) = 3.03$, $p_{(\text{one-tailed})} = .002$, $d_z = 0.51$.

There was not a significant *n* – 2 repetition cost following cue-only trials (-1.16%), $t(34) = -1.79$, $p_{(\text{one-tailed})} = .959$, $d_z = -0.30$, or following completed trials (-0.66%), $t(34) = -1.23$, $p_{(\text{one-tailed})} = .886$, $d_z = -0.21$. The *n* – 2 repetition cost following completed trials was not significantly larger than that following cue-only trials (0.50%), $t(34) = 0.53$, $p_{(\text{one-tailed})} = .300$, $d_z = 0.09$.

The main effect of trial sequence in the ANOVA was significant, $F(1,34) = 6.34$, MSE = 4.57, $p = .017$, $\eta_p^2 = .157$, with participants making 0.91% fewer errors on ABA trials (8.19%) than on CBA trials (9.10%). The main effect of trial *n* – 1 completion was not significant, $F(1,34) = 0.37$, MSE = 14.86, $p = .548$, $\eta_p^2 = .011$ (completed: 8.44%, cue-only: 8.84%). The interaction between trial sequence and trial *n* – 1 completion was not significant, $F(1,34) = 2.282$, MSE = 7.83, $p = .599$, $\eta_p^2 = .008$.

Discussion

In this experiment we varied the cue-target interval and showed that participants' overall performance benefitted from having more time in which to prepare the cued task. While there was a significant *n* – 2 repetition cost following completed trials, following cue-only trials there was neither a significant benefit nor a significant cost of *n* – 2 task-repetition.

While there was no significant *n* – 2 repetition cost following cue-only trials, the lack of a significant benefit is potentially consistent with there having been a small amount of inhibition applied to the preceding task on cue-only trials, just enough to overcome the facilitation resulting from that task having been recently completed (see evidence from computational modelling by Grange, Juvina, & Houghton, 2013). So, compared to Experiments 2 and 3, there may be more reason to allow for the possibility of proactively-driven backward inhibition having taken place in this experiment. However, the lack of a significant *n* – 2 repetition cost makes it difficult to be confident that backward inhibition took place.

In addition, with hindsight we were not satisfied that our measure of task preparation was specific enough to be able to draw strong conclusions. Since we did not control the total time between trials, the effect of cue-target interval on overall performance need not indicate use of the cue as such, but might instead simply reflect a passive effect of time having elapsed since the preceding trial. (N.B. Schuch & Koch's design did not have this limitation since it equated time between trials across cue-target interval conditions.) Hence, even if the absence of a significant *n* – 2 repetition cost did indicate that no backward inhibition had been triggered proactively, we would not have convincingly demonstrated that the conditions indicated that advance task preparation took place, and therefore would not strongly be able to conclude that task preparation was insufficient to trigger backward inhibition in this experiment.
